# Enhanced physician prompts in prenatal electronic medical records impact documentation on smoking cessation^*^

**DOI:** 10.4236/ojog.2013.310132

**Published:** 2013-12-01

**Authors:** Lisa D. Levine, Jitsen Chang, Irwin R. Merkatz, Peter S. Bernstein

**Affiliations:** 1 Department of Obstetrics and Gynecology, Maternal and Child Health Research Program, University of Pennsylvania Perelman School of Medicine, Philadelphia, USA; 2 Department of Obstetrics and Gynecology, Cedar Sinai Medical Center, Los Angeles, USA; 3 Department of Obstetrics and Gynecology and Women’s Health, Albert Einstein College of Medicine, Montefiore Medical Center, Bronx, USA

**Keywords:** Prenatal EMR, Physician Prompts, Smoking Cessation

## Abstract

**Objective:**

Smoking cessation during pregnancy is a modifiable intervention that can improve maternal and neonatal outcomes. Encouraging smoking cessation is an assessed measure of the Meaningful Use incentives to ensure best practices with the increased use of the electronic medical record (EMR). Physician EMR prompts have been used shown to be successful with preventive care but there is a paucity of data evaluating prompts within obstetrics. The objective of this study is to determine the effectiveness of enhanced smoking cessation prompts in a prenatal EMR.

**Methods:**

A retrospective cohort study of an enhanced smoking cessation prompting system within our prenatal EMR was performed. Pregnant women who reported tobacco use at first prenatal visit were included. The number of times a smoking cessation method was offered and documented, the number of documented attempts at smoking cessation, and the final number of cigarettes smoked were compared pre and post the enhancement of the smoking cessation prompting system.

**Results:**

95 patients were included (48 pre-enhancement; 47 post-enhancement). Post-enhancement, the documentation of smoking cessation method offered increased (0 vs. 1, p = 0.03) and documentation of smoking cessation attempts increased (1 vs. 2, p = 0.006). There was no change in the final number of cigarettes smoked (p = 0.9).

**Conclusions:**

Enhanced prompting systems increase documentation related to smoking cessation with no change in number of cigarettes smoked. In the era of Meaningful Use guidelines which focus on documentation in the EMR, continued research must be done to assure that software enhancements and improved documentation truly result in improved patient care.

## 1. INTRODUCTION

The increased risk of poor pregnancy outcomes among women who smoke is well known and the importance of smoking cessation during pregnancy is a goal emphasized by numerous professional and public health organizations [1–3]. In addition, efforts to encourage smoking cessation have become a measure to be reported to the federal government as part of Meaningful Use incentives designed to ensure best practices with the adoption of electronic medical records (EMR) by health care organizations [4].

While directed counseling sessions by trained professionals have been shown to be an effective approach for achieving smoking cessation among pregnant women, the data suggest that physicians must also offer effective tobacco cessation interventions throughout prenatal care [5,6]. Rigotti and colleagues demonstrated that although 80% of the pregnant women in their study were encouraged to quit smoking by their prenatal care provider, only 44% of them were offered a specific smoking cessation method [7].

In the wake of a changing healthcare system where the providers have less time with each patient, there may be an increasing role for reminders and prompts to encourage providers to address important issues during office visits. For example, studies have demonstrated that appropriate physician reminders and prompts result in improved delivery of preventive care in a variety of settings [8–11]. Most recently, Klatt *et al.* demonstrated effectiveness of administration of influenza vaccine during prenatal care with the use of physician prompts [12].

Given the new Meaningful Use reporting standards, it is important to understand the potential utility of prompting providers to address smoking cessation through the use of an EMR. The objective of this study, therefore, was to determine the effectiveness of an enhanced smoking cessation prompting system in our prenatal EMR. We hypothesized that this enhancement would lead to improved counseling and assistance with quitting methods, improved documentation of smoking status and cessation attempts, and increased success at overall smoking cessation.

## 2. METHODS

A retrospective cohort study was performed with approval from the Montefiore Medical Center Institutional Review Board. In 2003, a prenatal EMR (AS OBGYN, AS Software, Inc., Fort Lee, NJ) was implemented. At the first prenatal visit the EMR guides providers through a comprehensive list of questions to assist in obtaining a complete risk assessment for each patient. These include specific questions regarding patient tobacco use. A positive answer to any question creates an entry on the problem list for the patient in the EMR. This problem list is presented to the healthcare provider at each prenatal visit along with any notes that have been written in relation to that problem entry at any subsequent visit. In the initial version in 2003, the tobacco use questions only prompted the providers to ask how many cigarettes the women smoked prior to pregnancy and how many they were currently smoking. The documented responses to these prompts became a structured text note that was connected to the tobacco use entry on the patient’s problem list.

In 2006, the format of these questions were enhanced to further prompt the provider to advise the patient to quit, to assess the patient’s willingness to quit, and to offer a referral to the New York State Tobacco Quitline support program (Table 1). The structured text of these questions, along with the responses, also appears in the problem list entry.

Women who reported tobacco use at first prenatal visit and who continued their care at one of the Montefiore Medical Center sites that utilized the AS OBGYN software were included in the study. The providers at these sites include attending and resident physicians. Each woman may be seen for prenatal care by a variety of practitioners. Women must have initiated prenatal care in the first or second trimester in order to allow for multiple opportunities for providers to discuss smoking cessation. Patients who miscarried or terminated their pregnancies after their first prenatal visit or who transferred their prenatal care to a site outside of our institution were excluded.

Patients with an estimated delivery date (EDD) in 2005, the year immediately prior to the EMR changes, were chosen as the unexposed group, and patients with an EDD in 2007, one year after the enhanced tobacco prompts were implemented, were chosen as the exposed group. In all of the patients, tobacco use involved only cigarette smoking. No other changes to the prenatal risk assessment were made during this time period. No interval training was given to providers regarding counseling on smoking cessation or smoking cessation techniques during this time period. Patients were randomly selected during these two time periods.

Data collection included: age, parity, race, number of prenatal visits, number of cigarettes smoked prior to pregnancy, number smoked at the first prenatal visit, number of previous attempts at quitting, number of quitting attempts in pregnancy, number of years smoking, final number of cigarettes reported to have been smoked in pregnancy, amount of times provider documented smoking cessation status, whether or not a specific method for smoking cessation was offered, and smoking status at post partum visit.

Our primary outcome was the number of times a smoking cessation method was offered to a patient. Our secondary outcomes were the number of times the current smoking status was documented, the number of times a provider documented a patient’s attempt at smoking cessation, the number of cigarettes the patient cut down to, and the success of smoking cessation at post partum visit. Data were abstracted by two of the authors (LDL, JC) who received training with AS OBGYN software and had specific guidelines for detecting the outcomes. Given that the date of the prenatal visit is listed next to the problem list text, the abstractors were not able to be blinded to the exposure.

We assumed 50% of patients had a smoking cessation method offered and documented prior to the prompt enhancement. Using a 2-sided Type 1 error of 5%, and a power of 80%, we calculated that we needed 45 patients in each group to detect an increase in documentation to 80% after the prompt enhancement. All data were analyzed using Stata version 12.0 (College Station, TX). Categorical data including demographic information were compared using chi-square tests. Continuous data were analyzed utilizing two sample t-test and Mann-Whitney U test where appropriate. Statistical significance of p < 0.05 was used.

## 3. RESULTS

**Figure 1:**
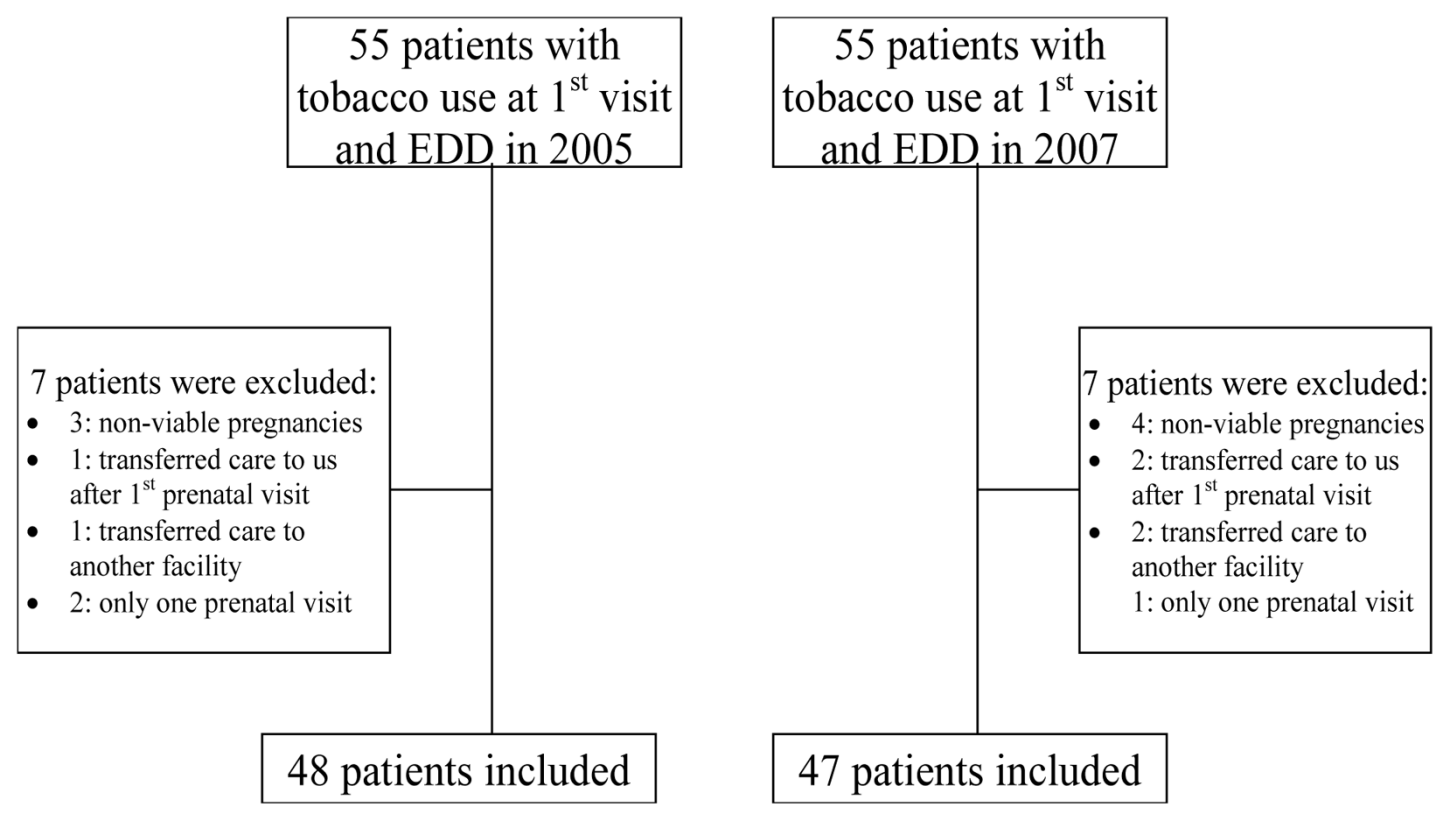
Flowchart of patients included in the study. EDD: Estimated Delivery Date.

We identified a total of 55 patients that had “tobacco use” documented at their first prenatal visit and had an EDD in 2005 and 55 patients with an EDD in 2007. Of those, 48 patients with an EDD in 2005 and 47 patients with an EDD in 2007met inclusion criteria (Figure 1).

The average age at first prenatal visit was 27.9 years. Of the 95 patients that were included in the analyses, 52% had a documented race with 34% African American, 53% Hispanic, and 12% Caucasian. Baseline maternal demographics between the pre and post enhancement groups were not significantly different (Table 2).

After the enhanced smoking cessation prompting system was introduced, the median number of times a cessation technique was offered and documented significantly increased, p = 0.03 (Table 3). Additionally, the number of times a provider documented a patient’s attempt at smoking cessation during pregnancy significantly increased after the prompt enhancement, p = 0.006. The median number of times the current smoking status was documented was not significantly different between the groups (p = 0.06). The median final number of cigarettes the patient reported to have cut down to was zero for both groups with no significant difference between the groups (p = 0.9), Table 3.

Of the 95 patients that were included in the study, 20 (21%) returned for a postpartum follow-up visit. There was no difference in the number of women who came for a postpartum visit between the pre and post enhancement groups (19% vs. 22%, p = 0.7). Among the 20 women who returned for a visit, 3 reported smoking at the postpartum visit, 4 reported continued cessation of smoking, and 13 had no mention of the smoking status. This was not significantly different between the two groups (p = 0.09).

## 4. DISCUSSION

Our study demonstrates a significant increase in the number of times a specific smoking cessation method was offered to patients and an increase in provider documentation of quitting attempts made by pregnant smokers after the implementation of an enhanced smoking cessation prompting system in our prenatal EMR. While these results are statistically significant, the clinical impact remains unclear.

Dexheimer *et al.* conducted a systematic review of randomized controlled trials that evaluated the use of physician prompts in preventive care. They found that computerized reminder systems were effective in increasing preventive care services most significantly in the delivery of cardiac care and smoking cessation [11]. This study was not performed in a pregnant population; however, the findings from their study are consistent with our results. Klatt *et al.* described an improvement in influenza vaccination among pregnant women with the use of physician prompts [12] which supports its use during prenatal care.

Our study has several strengths. To our knowledge, it is the first study to look at the use of physician prompts in addressing smoking status and smoking cessation during pregnancy. Pregnancy is a unique opportunity to capture a patient population that is motivated and may not otherwise seek medical care. Prompts reminding physicians to discuss specific methods of quitting allows for additional discussion of smoking status and increases counseling which is an important preventive care service. Additionally, our study drew from a large number of patients who were cared for at a number of diverse prenatal care sites by a variety of providers within an urban area. We minimized the effects of various secular trends in documentation that may have occurred in earlier years by choosing a time period immediately preceding the prompt enhancement. We also allowed for acclimatization by practitioners to use of the enhanced prompts by choosing a time period one year after the prompt enhancement was completed.

Our study was not without limitations. As a retrospective study, chart abstraction must be relied on to obtain all data. Therefore, it is difficult to ensure actual changes in practice and counseling versus changes in documentation alone that may have occurred after the prompt enhancement. For example, in 2005, it is not known whether or not a quitting method was offered to a patient but not documented in the chart since there was no EMR prompt reminding a provider to offer a method and document accordingly at that time. Additionally, we cannot exclude the possibility that other factors in the year between the two samples resulted in changes in provider documentation habits rather than the new enhanced prompts in the prenatal medical record system. Furthermore, our study was not powered to show a reduction in tobacco use by our patients, which could explain the non-significant difference in this outcome. Since the median number of cigarettes the patients cut down to was zero in both groups, this may be a low cigarette smoking population, further contributing to the explanation for not seeing a difference between the two groups. Therefore, a larger sample size might be needed to show a more robust difference between the groups. Lastly, the low postpartum visit rate makes it difficult to conclude the benefit these prompts have on smoking cessation after pregnancy.

## 5. CONCLUSION

Despite these limitations, our study is important for medical practice and prenatal care. Achieving smoking cessation during pregnancy is a modifiable intervention that can improve the outcome of pregnancy [2,3]. The intent of the government’s inclusion of smoking cessation documentation into their Meaningful Use Guidelines is to encourage providers to focus on this important area of public health. These Guidelines, which focus on documentation in the medical record, will be used to assess the care provided to patients [4]. An assumption exists that electronic medical records will result in improved documentation and better quality of care for patients. What is evident from our study is that these guidelines may lead to improved documentation; however, the clinical impact remains unclear. There remains a need for further investigation into the impact that improved documentation truly has on clinical care and patient outcomes. In the era of Meaningful Use Guidelines in which providers are asked to build certain functionalities into their EMR, continued research must be done to assure that improvements we observed in documentation will impact the rate of smoking cessation in our population.

## Figures and Tables

**Table 1:** Smoking cessation questions in EMR.

Number of cigarettes per day prior to pregnancy: Number of cigarettes per day during pregnancy: Did you advise to quit/remain abstinent?* Does the patient report they’re ready to quit?* Since, last visit, have they tried to quit?* Has a referral been made to the Quitline or other method of cessation offered?*

* New questions implemented in 2006.

**Table 2:** Maternal demographic information pre and post prompt enhancement.

	Pre-prompt enhancement (n = 48)	Post-prompt enhancement (n = 47)	p-value
Age, years*	26.9 (±6.7)	29.1 (±6.2)	0.1
**Race – n (%)**			
**AA**	11 (39)	6 (27)	0.2
**Hispanic**	12 (44)	14 (64)	
**White**	4 (14)	2 (9)	
Parity*	1.1 (± 1.6)	1.3 (± 1.4)	0.4
Gestational age at 1^st^ PNV (weeks)*	13.4 (± 5.4)	15.6 (± 6.6)	0.2
Total No. of PNV*	8.0 (± 3.8)	9.4 (± 4.4)	0.1
No. of prior attempts to quit**	1 (1 – 4)	1 (1 – 4)	0.2
No. of cigarettes prior to pregnancy**	10 (4.5 – 20)	7.5 (3 – 17.5)	0.4
No. of cigarettes at first PNV**	2 (0 – 3)	2 (0 – 5)	0.7

* Mean (±Standard deviation);

** Median (Interquartile range); AA: African American; PNV: prenatal visit; No: number.

**Table 3:** Results of smoking cessation pre and post prompt enhancement.

	Pre-prompt enhancement (n = 48)	Post-prompt enhancement (n = 47)	p-value
**No. of times cessation technique was offered**	0 (0)	1 (0 – 1)	0.03
**No. attempts to quit during pregnancy**	1 (0 – 3)	2 (0 – 5)	0.006
**No. of times the smoking status was documented**	1 (1 – 2)	1 (1 – 4)	0.06
**No. of cigarettes reported to have cut down to**	0 (0 – 3)	0 (0 – 3.5)	0.9

Numbers are presented as medians (Interquartile range); No: number.
